# Superior and Inferior Extension of Carotid Body Tumors

**DOI:** 10.4021/wjon485w

**Published:** 2012-07-05

**Authors:** Mohammad Esmadi, Humera Ahsan, Dina S Ahmad, Ruth GovierBrush

**Affiliations:** a Department of Internal Medicine, University of Missouri School of Medicine, Columbia, MO, USA; b Department of Radiology, University of Missouri School of Medicine, Columbia, MO, USA

**Keywords:** Carotid body tumor, Paraganglioma, Carotid body, Head and neck neoplasms, Angiography

## Abstract

Carotid body tumors typically arise at the level of the common carotid bifurcation where the carotid body exists. Superior and inferior extension beyond the carotid body may occur as well, especially if the tumor is long-standing. We herein describe a case of carotid body tumor in a patient who presented with a right-sided neck mass for 30 years. Computed tomography angiography (CTA) and B-mode sonography with color-coded Doppler sonography showed a vascular tumor arising at the level of the right common carotid artery bifurcation with superior and inferior extension beyond the bifurcation. This report emphasizes the utility of computed tomography angiography (CTA) and B-mode sonography with color-coded Doppler sonography as non-invasive modalities in visualizing the extension of carotid body tumors.

## Introduction

Paragangliomas are rare tumors that arise from widely dispersed specialized neural crest cells that are associated with autonomic ganglia. Carotid body tumors represent 60 - 70% of head and neck paragangliomas [1-2]. A carotid body paraganglioma arises within the carotid body and characteristically splays the bifurcation of the common carotid artery (CCA). Carotid body tumors can be diagnosed by B-mode sonography with color-coded Doppler sonography, computed tomography (CT), magnetic resonance imaging (MRI) and digital substraction angiography (DSA). Our case demonstrates an extension of carotid body tumor superior and inferior to the bifurcation of the common carotid artery beyond the main bulk of the tumor with emphasis on the role of computed tomography angiography (CTA) and B-mode sonography with color-coded Doppler sonography as non-invasive modalities in visualizing the extension of carotid body tumors.

## Case Report

We describe herein a case of a 53-year-old male who presented with a painless neck mass that he first noticed 30 years ago. The initially small mass has progressively grown and is now accompanied by night sweats and dizziness. The patient denies blurry vision, parasthesia, paralysis, palpitations, difficulty swallowing, or changes in voice.

Physical examination showed a right neck mass about 4 x 3 cm. It was non-tender, and does not move with swallowing. No lymphadenopathy was appreciated. Cranial nerves I-XII were grossly intact except for decreased sensation in his right lower mandible and the superior right aspect of his neck.

**Figure 1:**
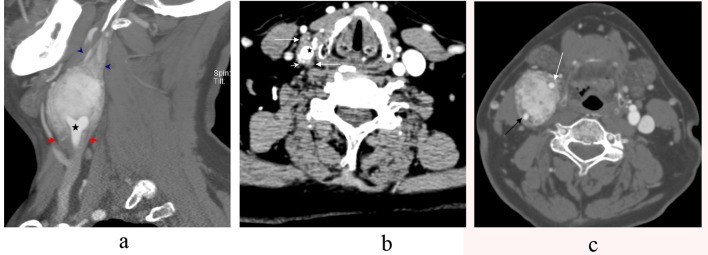
a-c. CT Angiography sagittal section showing a large carotid body tumor splaying the external carotid artery (ECA) and the internal carotid artery (ICA) at the level of th common carotid bifurcation (star). The tumor notably extends inferiorly 1.6 cm below the common carotid bifurcation (arrows). It also extends approximately 1.6 cm superiorly above the main bulk of the tumor (arrowhead) (a). Axial CT Angiography of the neck showing the tumor (long arrows) wrapping the common carotid artery (star) below the bifurcation with avid vascularity of the tumor (short arrows) (b). Axial CT Angiography section demonstrating an avidly enhancing mass splaying the internal carotid artery (black arrow) and the external carotid artery (white arrow) (c).

CT angiography of the neck demonstrated an avidly enhancing mass splaying the right external carotid artery (ECA) and the internal carotid artery (ICA). The mass begins 1.6 cm below the right CCA bifurcation with the bulk of the tumor measuring 4.3 x 3.4 x 5.8 cm. There was additional tumor projecting approximately 1.6 cm above the dominant mass. The right internal jugular vein is compressed (Fig. 1a-c).

**Figure 2:**
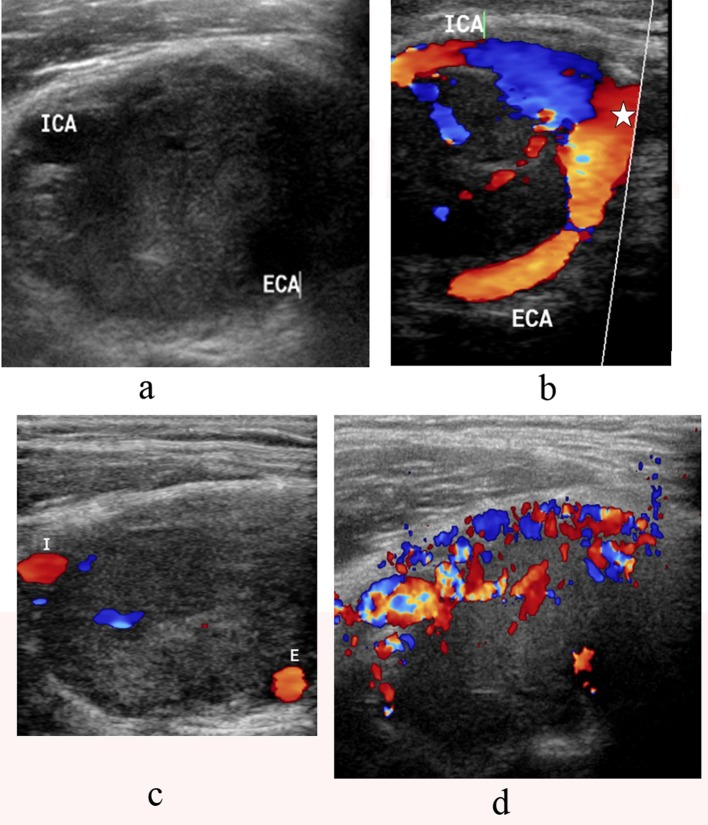
a-d. B-mode sonography showing a solid, well-defined, and hypoechoic tumor splaying the internal carotid artery(ICA) and external carotid artery (ECA) (a). Color flow Doppler sonography demonstrates a large mass at the carotid bifurcation. There is splaying of the internal carotid artery (ICA) and the external carotid artery (ECA) starting from the level of the common carotid bifurcation(star) (b, c) with high vascularity (d).

B-mode sonography showed a solid, well-defined, and hypoechoic tumor splaying the ICA and ECA (Fig. 2a). Color flow Doppler sonography showed also splaying of the internal and external carotid arteries by the mass with intratumoral vascularity (Fig. 2b-d).

## Discussion

Carotid body tumors can be diagnosed by B-mode sonography with color-coded Doppler sonography, computed tomography (CT), magnetic resonance imaging (MRI) and digital substraction angiography (DSA). B-mode sonography typically shows a solid, well-defined, and hypoechoic tumor. Color Doppler imaging in most cases, but not all, reveals intratumoral hypervascularity. B-mode sonography and color Doppler imaging can both display the splaying of the carotid bifurcation with displacement of the external carotid anteriorly and both the internal carotid and the internal jugular vein posteriorly [3-4].

CT Angiography is capable of showing the highly vascular tumor as a well-defined soft-tissue mass within the carotid space of the infrahyoid part of the neck with characteristic splaying of the carotid bifurcation. The homogeneous and intense enhancement following intravenous administration of contrast material is due to the underlying hypervascularity of the tumor. Splaying of the carotid artery bifurcation has classically been described on conventional angiography and is known as “Lyre” sign [5-7]. This sign has also been described on multi-detector computed tomography [8]. Superior extension of the tumor into the suprahyoid part of the neck is seen in 8% of the cases [9].

Digital Subtraction Angiography (DSA) is an invasive modality to diagnose carotid body tumors, but is more important for therapeutic purposes. It provides endovascular access for possible tumor embolization and identifies the blood supply and flow dynamics of the tumor. This would enable the surgeon to locate displaced vessels and identify more unpredictable and less accessible feeding vessels. Moreover, later phases of the arteriogram visualize the venous outflow, which provides information to the surgeons so that they can preserve the venous drainage until the final stages of tumor mobilization which results in decreased intraoperative blood loss [10].

In summary, ultrasound is a non-expensive, non-invasive modality that can be used as a first step to diagnose carotid body tumors. CT Angiography and MRI can show the extension of the tumor. Conventional angiography is an invasive modality for diagnosis, but is more important for embolization and preoperative evaluation. Our case emphasizes the utility of computed tomography angiography (CTA) and B-mode sonography with color-coded Doppler sonography as non-invasive modalities in visualizing the extension of carotid body tumors.
